# Clinical evaluation of marketed orthodontic products: are researchers behind the times? A meta-epidemiological study

**DOI:** 10.1186/s40510-017-0168-y

**Published:** 2017-05-25

**Authors:** Jadbinder Seehra, Nikolaos Pandis, Padhraig S. Fleming

**Affiliations:** 1 Department of Orthodontics, King’s College London Dental Institute, Floor 22, Guy’s Hospital, Guy’s and St Thomas NHS Foundation Trust, London, WC2R 2LS UK; 2 Department of Orthodontics and Dentofacial Orthopedics, Dental School/Medical Faculty, University of Bern, Freiburgstrasse 7, Bern, CH-3010 Switzerland; 3 Barts and The London School of Medicine and Dentistry, Institute of Dentistry, Queen Mary University of London, Queen Mary University of London, London, E1 2AD UK

## Abstract

**Background:**

The role of marketing and industry in the treatment decisions of orthodontists has received increasing attention in recent years with clinical research typically undertaken subsequent to established use of these devices and often failing to confirm the promise of manufacturers’ claims. This meta-epidemiological study was undertaken to assess the proportion of clinical trials in orthodontics evaluating commercially marketed products and to evaluate the direction of the results of these studies.

**Methods:**

Electronic searching was undertaken to identify randomized controlled trials (RCTs) published over a 5-year period (1 January 2012 to 31 December 2016). Data obtained included the type of marketed intervention, direction of effect and declaration of both industry sponsorship and conflict of interest.

**Results:**

Eighty-four RCTs published in 23 scientific journals were included with the highest percentage in the *American Journal of Dentofacial Orthopedics* (AJO-DO) (23.8%), followed by the *European Journal of Orthodontics* (EJO) (14.3%), *Journal of Orthodontics* (JO) (10.7%) and *Angle Orthodontist* (AO) (10.7%). Overall, 45% (38/84) of clinical trials assessed involved analysis of marketed products after their introduction. Interventions to improve oral health or circumvent the risk of iatrogenic damage, such as white spot lesions, were most commonly assessed (15.8%), with the relative merits of non-surgical adjuncts (14.1%) and other orthodontic auxiliaries (13.1%) also frequently evaluated. In 44% of RCTs, a positive effect of the marketed intervention was not reported. Industry sponsorship of the research was declared in 9.5% RCTs. No significant associations between the direction of the effect and both declaration of industry sponsorship (*p* = 0.56) and conflict of interest (*p* = 0.96) were detected. Moreover, for marketed and non-marketed products, no significant associations for both declaration of industry sponsorship (*p* = 0.44) and conflict of interest (*p* = 0.28) were found.

**Conclusions:**

Almost half of orthodontic clinical trials over the past 5 years involve analysis of marketed products after their introduction. The results highlight a potential source of waste in orthodontic research emanating from existing approaches to licensing and marketing of orthodontic products.

## Background

Engagement of orthodontists with industry and commercial interests is necessary to facilitate the development, refinement and adoption of novel products. There has, however, been concerns in recent years that financial interests have led to vociferous advertisement and early adoption of relatively untested products, culminating in a plea that “truth not product should drive progress” in orthodontics [1]. This situation is not unique to orthodontics with an acceptance that clinical judgments relating to selection of medical pharmacological agents may be affected by inducements and financial motives [2, 3].

In order to mitigate the misleading effects of the fevered promotion of apparently unique products by commercial interests, clinical decisions should where possible be evidence-based. Ideally, the latter should be founded upon patient wishes and professional experience, allied to the assurance offered by best available evidence. While dentistry and orthodontics lagged behind pioneering medical specialties, evidence-based orthodontics is now firmly recognized [4]. In 2015, for example, there were 89 dental journals and eight orthodontic journals with an impact factor, with 596 citable papers published in these orthodontic journals alone, and the past decade has seen significant increases in the quantity of systematic reviews in orthodontics with 157 published between 2000 and 2014, although the yield from many of these has been limited [5].

Within this evidence-based approach, clinical trials which incorporate random allocation of participants to treatment groups are considered optimal to allow evaluation of the comparative effectiveness of interventions. However, clinical trials are expensive to undertake, are time-consuming and are demanding in terms of evaluation and follow-up. As such, while the relative frequency of clinical trials has increased in orthodontics, it is known that systematic reviews comparing the effectiveness of interventions almost invariably cite a lack of primary studies, particularly those of high quality, with a mean of just four clinical trials included in orthodontic meta-analyses [5]. Moreover, the spotlight has been placed on deficient conduct and reporting of biomedical research in the recent years [6]. In addition, meta-epidemiological research in orthodontics has indicated that inadequate randomization procedures, blinding and handling of missing data are pervasive within clinical trials [7].

However, in view of the marketing of orthodontic products and the lack of need for clinical evidence prior to advertisement and clinical adoption of novel orthodontic products, a further potential problem is the costly and avoidable focus of orthodontic trials on the evaluation of established, marketed products. This study therefore aims to assess the prevalence of clinical trials in orthodontics evaluating commercially marketed products. A further aim is to evaluate the direction of the results of these studies and whether these are affected by industry sponsorship.

## Methods

Electronic searching of a single database (PubMed) was undertaken in 15th February 2017. The term “orthodontics” was searched using PubMed filters. All English language randomized controlled trials (RCTs) in orthodontics over a 5-year period (1 January 2012 to 31 December 2016) were considered for inclusion. Based on the Cochrane criteria for the selection of RCTs, studies were screened for eligibility using the following eligibility criteria: human participants, interventions related to healthcare, experimental studies, presence of a control group and randomization of participants to control and treatment groups. Studies described in the title or abstract as “prospective”, “comparative”, or “efficacy” were further analysed to determine if randomization of participants was undertaken. Conference abstracts and laboratory-based randomized trials involving extracted human teeth were excluded. A single author (JS) screened potentially relevant articles.

All data were extracted using a pre-specified data collection sheet with specific coding of items. Data obtained from each study included the date of publication, journal, region of authorship (1 = Europe, 2 = America, 3 = other), number of authors, type and justification of marketed intervention, direction of intervention effect (1 = positive effect compared to control, 2 = negative effect compared to control, 3 = no difference detected between interventions or between intervention and untreated control) and declaration of both any industry sponsorship (1 = industry funded and declared, 2 = no industry sponsorship to declare, 3 = not clearly declared) and conflict of interest (1 = conflicts exist and declared, 2 = no conflicts to declare, 3 = not clearly declared).

### Statistical analysis

Descriptive statistics including means were used to report the study outcomes. Cross-tabulation between individual RCTs and marketed and non-marketed products reported direction of effect and declaration of industry sponsorship, and conflict of interest was undertaken using the chi-square test or Fisher’s exact *t* test as appropriate. The level of statistical significance for all tests was pre-specified at 0.05. Statistical analyses were performed with STATA® version 14.2 software (Stata Corporation, College Station, TX, USA).

## Results

**Fig. 1:**
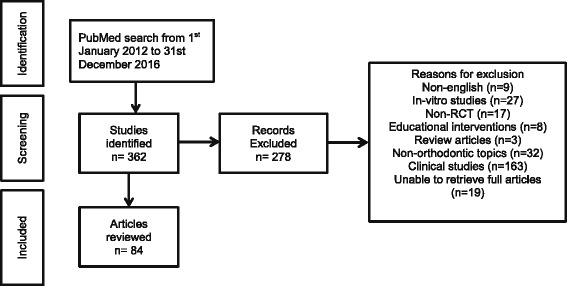
Study selection flowchart

Initial screening yielded 362 potentially relevant articles. Based on availability of the papers and the eligibility criteria, 278 results were excluded. In total, 84 RCTs published in 23 scientific journals were therefore included in this study (Fig. 1).

In terms of order of prevalence of RCTs within specific journals, the highest percentage were published in the *American Journal of Orthodontics and Dentofacial Orthopedics* (AJO-DO) (23.8%), followed by the *European Journal of Orthodontics* (EJO) (14.3%), *Journal of Orthodontics* (JO) (10.7%) and *Angle Orthodontist* (AO) (10.7%) (Table 1). The frequency of publication of RCTs per year was 2015 (27.4%), 2013 (23.8%), 2012 (22.6%), 2014 (19.0%) and 2016 (7.2%). The mean number of authors was 5.1 (SD 2.6, range 1–14). The majority of RCTs were conducted in Europe (48.1%), followed by non-American and European countries (33.3%) and American (17.9%).

**Table 1:** Characteristics of included randomized clinical trials (*N* = 84)

	*N*	%
Journal title		
*American Journal of Dentofacial Orthopedics* (AJO-DO)	20	23.8
*Angle Orthodontist* (AO)	9	10.7
*Australian Orthodontic Journal* (AOJ)	2	2.4
*Brazilian Oral Research* (BOR)	1	1.2
*Clinical Oral Investigations* (CIO)	2	2.4
*European Journal of Orthodontics* (EJO)	12	14.3
*European Journal of Oral Sciences* (EJOS)	1	1.2
*European Journal of Paedatric Dentistry* (EJPD)	1	1.2
*Journal of American Dental Association* (JADA)	1	1.2
*The Journal of Contemporary Dental Practice* (JCDP)	1	1.2
*Journal of Cranio-Maxillo Facial Surgery* (JCMFS)	1	1.2
*Journal of Clinical Periodontology*	1	1.2
*The Journal of Clinical Pediatric Dentistry*	1	1.2
*Journal of Dental Research*	4	4.8
*Journal of Orthodontics*	9	10.6
*Journal of Oral and maxillofacial Surgery*	2	2.4
*Journal of Orofacial Orthopedics*	1	1.2
*Lasers in Medical Science*	2	2.4
*Orthodontics and Craniofacial Research*	5	5.9
*Pediatric Dentistry*	1	1.2
*Progress in Orthodontics*	5	5.9
*PLoS ONE*	1	1.2
*Photomedicine and Laser Surgery*	1	1.2
Continent		
Europe	41	48.8
America	15	17.9
Non-European or American	28	33.3
Number of authors		
1–3	26	30.95
4–6	41	48.80
>7	17	20.25

Overall, 45% (38/84) of clinical trials assessed involved analysis of marketed products after their introduction. The remaining trials (46/84) assessed non-clinical interventions and non-marketed products such as growth modification and removable appliances, closing loops and archwires. Interventions to improve oral health and to circumvent risks of treatment, such as the treatment of post-orthodontic white spot lesions (15.8%), were most commonly assessed, with trials also frequently involving assessment of non-surgical adjuncts (14.1%) and orthodontic auxiliaries (13.1%). The most commonly cited justifications for the intervention under investigation were to promote treatment efficiency (33.3%), to reduce iatrogenic effects (20.2%) and to reduce pain experience during treatment (16.7%). In over 50% RCTs, a positive effect of the intervention was reported. Industry sponsorship of the research was declared in 9.5% RCTs. However, in a third (33.3%) of RCTs, whether industry sponsorship was involved is not clearly stated. The absence of a conflict of interest was clearly stated in 50%, while in 46.4%, whether a conflict of interest existed was not outlined (Table 2). For individual RCTs, no significant associations between the direction of the intervention effect and both declaration of industry sponsorship (*p* = 0.56) and conflict of interest (*p* = 0.96) were detected. Similarly, for marketed and non-marketed products, no significant associations for both declaration of industry sponsorship (*p* = 0.44) and conflict of interest (*p* = 0.28) were found.

**Table 2:** Results for primary and secondary outcomes

Category	*N*	%
Type of product		
Marketed products	38	45
Non-marketed products	46	55
Intervention		
Orthodontic bracket	8	9.5
Orthodontic archwire	2	2.4
Removable appliance	5	5.9
Non-surgical adjunctive	12	14.1
Surgical adjunctive	4	4.8
Retention	5	5.9
Oral health	13	15.8
Orthodontic auxiliaries	11	13.1
Interceptive treatment	5	5.9
Materials	8	9.5
Growth modification	7	8.3
Medication	4	4.8
Justification of intervention		
Accelerate treatment	7	8.3
Aesthetics	2	2.4
Reduce iatrogenic effects	17	20.2
Retain tooth position	5	5.9
Reduce pain	14	16.7
Improve knowledge	1	1.2
Oral health	3	3.6
Dental development	5	5.9
Treatment efficiency	28	33.3
Compliance	2	2.4
Direction of effect		
Positive	46	54.8
Negative	1	1.2
No difference	37	44.0
Industry sponsorship		
Industry funded and declared	8	9.5
No industry sponsorship to declare	48	57.2
Not clearly declared	28	33.3
Conflict of interest		
Conflicts exist and declared	3	3.6
No conflicts to declare	42	50
Not clearly declared	39	46.4

## Discussion

The present meta-epidemiological study highlights that almost half of orthodontic trials involve clinical evaluation of the relative merits of marketed products. Many of these do not confirm the effectiveness of these interventions, with almost 44% reporting no improvement related to the product. While these findings do not detract from the centrality of developing novel interventions in enhancing and streamlining orthodontics, it does suggest that there may be a disconnect between the marketing and clinical outcomes. This also highlights the potential merit in incorporating independent clinical research earlier in the research and development process [8].

A relatively high percentage of contemporary clinical trials (one third) focused on techniques to hasten orthodontic treatment—a zeitgeist in contemporary practice. These include both and non-surgical adjuncts with these thus far offering limited, largely equivocal results. Moreover, little difference in the rate of tooth movement with both surgical approaches and non-surgical approaches has been highlighted in recent systematic reviews—the latter encompassing vibratory stimulation, use of masticatory adjuncts and light-mediated aids [9, 10]. Despite the relatively high number of primary studies identified in the present review, these systematic reviews have bemoaned a lack of high-quality evidence within these areas suggesting that further research may still be required. The onus remains, therefore, on undertaking clinical trials with low risk of bias and focusing on consistent and relevant outcomes [11]. Furthermore, non-surgical adjuncts have associated cost and often rely on patient compliance. There is also evidence that surgical adjuncts may lead to anxiety and potentially hamper experiences of treatment [12].

A considerable percentage of studies involved assessment of interventions to reduce pain during orthodontics and other harms associated with treatment. This focus is encouraging as these outcomes are likely to resonate both with patients and professionals with pain known to represent a common reason for avoidance and indeed abandonment of orthodontic treatment [13]. Novel approaches evaluated include both pharmacological and novel non-pharmacological agents with increasing emphasis on the latter, although evidence in support of a range of non-pharmacological agents, including vibratory stimulation and masticatory adjuncts, in reducing subjective pain experience is limited [14].

Overall, the findings from the present review place existing practices in terms of the licensing and marketing of orthodontic devices and products into focus. In particular, it is accepted that clinical evidence evaluating the relative merits of a clinical technique follow its introduction. As such, the licensing of these techniques does not hinge on the existence of this supporting clinical data. Historically, the potential pitfalls associated with this approach were exemplified by the re-introduction of self-ligating brackets. This was accompanied by positive and indeed compelling marketing, which was given credence by the findings from early non-randomized studies [15, 16]. However, these observations were contradicted by more robust randomized studies, which consistently failed to corroborate either the manufacturers’ claims or the findings from earlier research [17, 18]. This situation is typical of differences in research design [19] and was aggravated by the subsequent refinement of bracket sub-types, including introduction of updated versions, meaning that by the time potentially influential research was published, obsolete products were being reported on. As such, clinical research should be encouraged at an earlier stage during orthodontic product development to mitigate against this time lag and to provide a meaningful and contemporary clinical underpinning.

There is no doubt that industry has facilitated the refinement of orthodontic practices and experiences with relatively recent advancements including the advent of fully customized labial and lingual systems, as well as very meaningful change in appliance aesthetics [20]. Many of these improvements are clear and do not necessarily require supporting evidence; however, claims in relation to reduced treatment times and lower requirement for extractions, for example, both require clinical evidence. In the absence of evidence in this respect, advertising claims may be misleading both to clinicians and patients, as evidenced in the medical literature [21, 22], particularly when marketed directly to the latter. As such, it is important for both that this is sought at an early juncture but also that advertising claims are modified and, where necessary, moderated in response to emerging clinic evidence. Enforcement of the latter could certainly be undertaken in advertisements appearing in dental publications and at dental conferences. Indeed, an analysis of advertisements in orthodontic journals over a 2-year period (2012–2013) highlighted that many of the claims made are not evidence-based with one quarter of these citing unpublished research [23]. Moreover, given the recent emphasis on poor yield from biomedical research [6], it is important to highlight that there is an academic cost attached to clinical evaluation of marketed products after their development, with the research itself being costly and time-consuming. In addition, this approach has the potential to stymie researchers from themselves undertaking original research and development, while focusing on secondary evaluation. As such, proving the clinical effectiveness of a product or in some cases debunking false marketing claims may have negative implications on the overall trajectory of orthodontic research. This is particularly important at present in view of the high proportion of dental research that is devoid of patient-centred outcomes, potentially further diluting the benefit and relevance of clinical research [24]. At a time when academic research funding is constrained, funding of independent academics by industry may therefore be encouraged to mitigate this; examples of this are beginning to emerge [25]. Notwithstanding this, it is important that these funded studies are undertaken and reported independently; meta-epidemiological findings from restorative dentistry suggest that best practice is being followed in this respect at present [26].

The present study was restricted to clinical trials, in isolation. Moreover, relatively few clinical trials were identified; however, we did obtain a sample over a 5-year period. It is therefore likely representative of contemporary research practice. Further analysis could be undertaken in time; however, unless changes are made in relation to regulatory requirements or orthodontic companies make a decision to embrace independent clinical research at an earlier stage, it is likely that the status quo will continue to apply.

## Conclusions

Overall, 45% (38/84) of orthodontic trials over the past 5 years have involved analysis of marketed products after their introduction. Many of these have focused on techniques to accelerate tooth movement and to reduce both orthodontic pain and other side effects. The results highlight a potential source of waste in orthodontic research emanating from existing approaches to licensing and marketing of orthodontic products.
